# Supplementary material for: Condom use increased after a peer group intervention implemented by community volunteers in Malawi
Source: BMC Public Health. 2024 Jun 3;24:1483. doi: 10.1186/s12889-024-18991-z (PMC11145788; doi:10.1186/s12889-024-18991-z)
Supplement: Supplementary file 1 — Additional File 1: Demographic and psychosocial factors for the total participants. (Additional File 1.pdf). This file contains the frequencies for all covariates for the total participants at Time 1 (baseline) and also at Times 2 and 3 for time-varying covariates [file 12889_2024_18991_MOESM1_ESM.docx]

**Additional File 1. Demographic and psychosocial factors for**

**the total participants**

|  | **Total**  **(Baseline N = 1008)** |
| --- | --- |
| **Baseline factors:** |  |
| **Community, N (%)** |  |
| Community 1 | 338 (33.5%) |
| Community 2 | 335 (33.2%) |
| Community 3 | 335 (33.2%) |
| **Gender, N (%)** |  |
| Female | 514 (51.0%) |
| **Age Group** |  |
| Adult | 460 (45.6%) |
| **Age, Mean (SD)** | 24.5 (12.0) |
|  |  |
| **Education, N (%)** |  |
| Did not complete primary school | 493 (48.9%) |
| Completed primary school | 370 (36.7%) |
| Completed secondary school | 145 (14.4%) |
| **Involvement in religiously affiliated activities, N (%)** |  |
| Very involved | 650 (64.5%) |
| **Time-varying covariates:** |  |
| **Partner Status, N (% married or cohabiting)** |  |
| T1 | 436 (43.3%)  N=1008 |
| T2 | 395 (42.7%)  N=926 |
| T3 | 465 (50.4%)  N=922 |
| **UNAIDS Knowledge, N (%)** |  |
| T1 | 429 (42.6%)  N=1008 |
| T2 | 471 (50.9%)  N=926 |
| T3 | 521 (56.5%)  N=923 |
| **Safer sex self-efficacy Index, Mean (SD)** |  |
| T1 | 10.7 (1.7)  N=1008 |
| T2 | 10.9 (1.6)  N=925 |
| T3 | 11.0 (1.4)  N=923 |
| **Partner communication index,**  **Mean (SD)** |  |
| T1 | 2.8 (1.2)  N=600 |
| T2 | 2.8 (1.2)  N=610 |
| T3 | 2.9 (1.1) N=661 |
